# Effects of Nasal Packing on Patients’ Post-operative Vital Signs

**DOI:** 10.7759/cureus.62616

**Published:** 2024-06-18

**Authors:** Saleh Khurshied, Muhammad A Zahid, Aisha Babar, Muhammad Hamza Rafique, Nawal Khurshid, Altaf Hussain, Mahrukh Saif

**Affiliations:** 1 Otolaryngology - Head and Neck Surgery, Pakistan Institute of Medical Sciences, Islamabad, PAK; 2 Ophthalmology, Monash Health, Clayton, AUS

**Keywords:** post-operative, nose surgery, hr: heart rate, spo2 saturation of oxygen in blood, high blood pressure

## Abstract

Background

Since bilateral nasal packing entails nasal and airway obstruction, this practice consequently leads to oral breathing. The resulting hypoxemia may then negatively impact vital signs, including blood pressure (BP), blood oxygen saturation (SpO2), and heart rate (HR). These systemic effects have a detrimental effect on patients.

Objective

The objective of this study is to observe the effects of bilateral nasal packing on patients’ post-operative vital signs.

Materials and methods

This prospective study was conducted in the department of otolaryngology - head and neck surgery over a six-month period. The study included 83 post-operative patients with nasal surgery, in which bilateral merocele nasal packing was performed. The patients’ pulse oximetry, systolic and diastolic BP, and HR were recorded four times the night before and after surgery. A statistical analysis was performed, and the mean values, standard deviation, and range were calculated. A paired sample t-test was also applied. The results are presented in figures and tables.

Results

The mean age of the participants was 27.65 ± 10.72 years, and 56 (67.5%) were male. Septoplasty was the most common surgery performed, with 63 participants having undergone this procedure (75.9%). When the pre-operative mean values of systolic and diastolic BP, SpO2, and HR were compared with the post-operative mean values, when a bilateral nasal pack was in place, a significant increase was found in all, with a p-value of <0.001 in each.

Conclusion

Bilateral nasal packing affects patients’ vital signs by significantly increasing diastolic and systolic BP and decreasing SpO2. The HR is also significantly increased when packing is in place.

## Introduction

The nose not only serves as an airway but also plays multiple roles, including filtering, moistening, and warming the inhaled air [1]. Normally, the nose itself contributes to half of the airway resistance, and any obstruction can increase this resistance [2,3]. Given the physiology of the nose, the nasal mucosa undergoes rhythmical, cyclical congestion and decongestion, thus controlling airway resistance through each nasal cavity [4]. An increased nasal resistance results in oral breathing, which affects respiratory mechanics and leads to hypoxemia, an alteration in arterial blood gases, and respiratory function tests [5,6]. After any nasal surgery, nasal packing is used as a standard procedure to stop bleeding by effectively pressing the walls of the nasal cavity, thus applying pressure and promoting hemostasis [7]. The practice also reduces the possibility of complications such as adhesions and septal hematoma. Although nasal packing stabilizes the septum and thus reduces the risk of post-operative deviation [8], this practice can also cause hypoxemia and hypercapnia [9]. Furthermore, since nasal packing causes nasal obstruction, the resulting obligatory mouth breathing may have systemic effects such as insomnia, breathing difficulty, decreased oxygen levels in the blood, and toxic shock syndrome [10].

This study argues that by having a deeper understanding of the systemic effects of nasal packing on patients’ vital signs, healthcare workers can improve how they manage patients and keep them comfortable post-operatively. Although otorhinolaryngologists usually know the local effects and complications of nasal packing, they often underestimate its systemic effects, which may cause morbidity for the patient. The objective of the study is to determine the effect of bilateral nasal packing on patients’ post-operative vital signs.

## Materials and methods

This prospective research was conducted in the department of otolaryngology - head and neck surgery at the Pakistan Institute of Medical Sciences (PIMS), Islamabad, from November 20, 2023, to April 20, 2024, for a duration of six months. The ethical clearance was granted by the Ethical Research Review Board of PIMS (approval number: F.3-1/2023(ERRB)/Chairman, approval date: November 17, 2023).

This study selected 83 patients, irrespective of gender, using consecutive convenient sampling techniques. The participants included were 15-75 years old and were undergoing their first nasal surgery. Bilateral anterior nasal packing of Merocel (a self-expanding, nonabsorbable dehydrated sponge) was also used for these patients, and the packing was removed the day after surgery. Potential participants who had any cardiopulmonary disease or any co-morbid conditions or needed any supplemental oxygen pre- or post-operatively were excluded from the study.

All patients were given standard, similar analgesics post-operatively. The patient’s blood oxygen saturation (SpO2) was measured by a digital pulse oximeter, their systolic and diastolic blood pressure (BP) were measured by a manual mercury sphygmomanometer by the on-duty doctor, and their radial pulse heart rate (HR) was measured by the on-duty doctor. These measurements were recorded the night before surgery and the night after surgery, when the bilateral nasal pack was in place. Four readings were taken with the same gap interval, and the mean was recorded. All information was collected on a proforma.

Standard statistical procedures and analysis were conducted using SPSS Statistics version 29.0 (IBM Corp. Released 2023. IBM SPSS Statistics for Windows, Version 29.0.2.0 Armonk, NY: IBM Corp). Frequencies, percentages, mean, standard deviation, and range were calculated. For qualitative variables such as gender and the type of surgeries performed, the frequency was calculated. All continuous variables were displayed as the mean value ± standard deviation and are presented in the form of a table. Numbers with percentages are displayed where required in the form of tables and figures. The paired sample t-test was used to compare the mean values of vital signs pre- and post-operatively. A p-value of less than 0.05 was considered significant.

## Results

**Figure 1:**
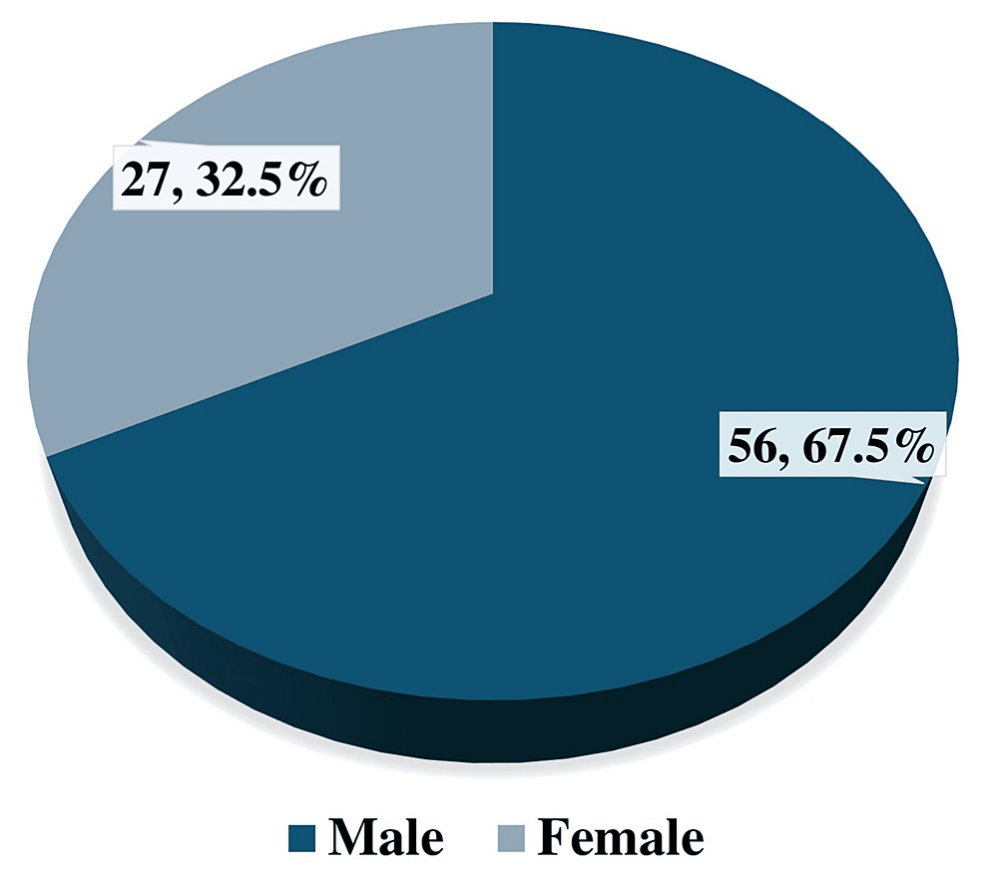
Gender distribution of participants

**Figure 2:**
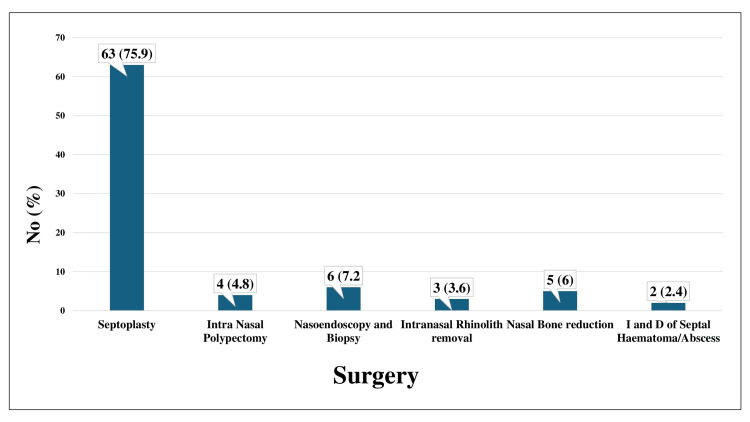
Distribution of participants based on surgeries for which nasal packing was performed

The mean age of the participants ± SD was 27.65 ± 10.72 years, with an age range of 16-66 years. The participants were divided into groups based on age, which is detailed in Table 1. Figure 1 presents the gender distribution, and the majority of the participants are male. Figure 2 indicates the distribution of different types of surgeries for which nasal packing was post-operatively performed. When the mean pre-operative systolic BP value was compared with the mean post-operative systolic BP value, a significant association was found between the two (p≤0.001). Similarly, when the mean pre-operative diastolic BP value was compared with the mean post-operative diastolic BP value, a significant association was found between the two (p≤0.001). When the mean of the pre-operative SpO2 value was compared with the mean post-operative SpO2 value, a significant association was found between the two (p≤0.001). Table 2 indicates that a significant association was found between the pre- and post-operative mean HR (p≤0.001).

**Table 1:** Age distribution of participants

Age group	No (%)
15-25	44 (53)
26-35	28 (33.7)
36-45	5 (6.0)
46-55	2 (2.4)
56-65	3 (3.6)
66-75	1 (1.2)

**Table 2:** Correlation between pre- and post-operative vital signs Pre-op: pre-operative, Post-op: post-operative, BP: blood pressure, mmHg: millimeter of mercury, SpO2: blood oxygen saturation, HR: heart rate, BPM: beats per minute

Vitals	Mean value ± SD	Range	p-value
Pre-op systolic BP (mmHg)	112.06 ± 9.84	90-132	<0.001
Post-op systolic BP (mmHg)	120.57 ± 9.16	98-136	
Pre-op diastolic BP (mmHg)	72.54 ± 6.71	56-90	<0.001
Post-op diastolic BP (mmHg)	77.72 ± 6.34	61-91	
Pre-op SpO2 (%)	97.52 ± 1.02	95-99	<0.001
Post-op SpO2 (%)	95.31 ± 1.47	93-99	
Pre-op HR (BPM)	76.07 ± 8.91	54-95	<0.001
Post-op HR (BPM)	86.03 ± 10.34	63-107	

## Discussion

Our study found that bilateral nasal packing can significantly increase the mean diastolic and systolic BP, decrease the mean SpO2, and increase the HR. We also compared our results with past research in the literature. Guan et al. found that bilateral nasal packing could cause sleep hypoxemia, which aligns with the results of our study, and patients with bilateral nasal packing had significantly low SpO2 levels post-operatively [11].

The study by Bista concluded that bilateral nasal packing could result in a significant rise in systolic and diastolic BP (p<0.001) and a decrease in SpO2 (p<0.001), and these results were similar to our findings [12]. Moreover, Turhan et al. found that nasal packing has a negative effect on respiration while sleeping and causes a significant decrease in oxygen saturation, and these findings are comparable to our results and methodology [13]. Similarly, another study showed that the risk of respiratory distress is 3.6 times greater in patients with nasal packing post-operatively [14]. Relatedly, Surrender et al. found that patients with nasal packing had a significant decrease in SpO2 post-operatively [15]. Their mean value of SpO2 was 98.3 ± 0.794% pre-operatively and 97.17 ± 1.744% 24 hours after surgery, which was comparable to our study.

In our study, 56 (67.5%) patients were male, while 27 (32.5%) of the patients were female. The mean age of the participants was 27.65 years, and these demographics are comparable to previously conducted studies [11-16]. The most common surgery for which nasal packing was performed for patients in our study was septoplasty, which matches the findings of other past studies [12,17]. Furthermore, Ogretmenoglu et al. concluded that nasal packing causes a significant increase in mean HR, similar to our findings where nasal packing significantly increases the HR [17]. Kiely et al. concluded that hypoxia due to nasal packing can lead to changes in HR, which may also lead to arrhythmias [18].

The limitations of our study were the small sample size, the data from a single hospital, and the comparison of different surgeries together. We also only compared mean values. We recommend that further research should assess a large sample size and be multicentric. Future studies should also consider other factors that can change the vital signs post-operatively and affect the results, and the lowest and highest values of vital signs should also be compared.

## Conclusions

Bilateral nasal packing causes a significant increase in diastolic and systolic BP and a decrease in SpO2. It also significantly increases the HR. These findings indicate that bilateral nasal packing does have significant systemic effects on the vital signs of patients, and these systemic effects should be kept in mind when bilateral nasal packing is placed, especially for high-risk patients where it can cause morbidity.
